# Assessment of the Impact of Using a Reference Transcriptome in Mapping Short RNA-Seq Reads

**DOI:** 10.1371/journal.pone.0101374

**Published:** 2014-07-03

**Authors:** Shanrong Zhao

**Affiliations:** Systems Pharmacology and Biomarkers, Janssen Research & Development, LLC, San Diego, California, United States of America; CNRS UMR7622 & University Paris 6 Pierre-et-Marie-Curie, France

## Abstract

RNA-Seq has become increasingly popular in transcriptome profiling. The major challenge in RNA-Seq data analysis is the accurate mapping of junction reads to their genomic origins. To detect splicing sites in short reads, many RNA-Seq aligners use reference transcriptome to inform placement of junction reads. However, no systematic evaluation has been performed to assess or quantify the benefits of incorporating reference transcriptome in mapping RNA-Seq reads. In this paper, we have studied the impact of reference transcriptome on mapping RNA-Seq reads, especially on junction ones. The same dataset were analysed with and without RefGene transcriptome, respectively. Then a Perl script was developed to analyse and compare the mapping results. It was found that about 50–55% junction reads can be mapped to the same genomic regions regardless of the usage of RefGene model. More than one-third of reads fail to be mapped without the help of a reference transcriptome. For “Alternatively” mapped reads, i.e., those reads mapped differently with and without RefGene model, the mappings without RefGene model are usually worse than their corresponding alignments with RefGene model. For junction reads that span more than two exons, it is less likely to align them correctly without the assistance of reference transcriptome. As the sequencing technology evolves, the read length is becoming longer and longer. When reads become longer, they are more likely to span multiple exons, and thus the mapping of long junction reads is actually becoming more and more challenging without the assistance of reference transcriptome. Therefore, the advantages of using reference transcriptome in the mapping demonstrated in this study are becoming more evident for longer reads. In addition, the effect of the completeness of reference transcriptome on mapping of RNA-Seq reads is discussed.

## Introduction

In recent years, RNA-Seq has become a popular and powerful approach for transcriptome profiling [1]–[6]. RNA-Seq not only has considerable advantages for examining transcriptome fine structure–for example, in the detection of novel transcripts, allele-specific expression, and alternative splicing–but also provides a far more precise measurement of levels of transcripts than that of other methods such as microarray [7]–[10]. Previously we had performed a side by side comparison of RNA-Seq and microarray in investigating T cell activation, and demonstrated that RNA-Seq is superior in detecting low abundance transcripts, differentiating biologically critical isoforms, and allowing the identification of genetic variants [7]. In addition, RNA-Seq also has a much broader dynamic range than microarray, which allows for the detection of more differentially expressed genes with higher fold-change. Furthermore, RNA-Seq avoids technical issues in microarray related to probe performance such as cross-hybridization, limited detection range of individual probes, and nonspecific hybridization. And thus, RNA-Seq delivers unbiased and unparalleled information about the transcriptome and gene expression [10]. Currently, RNA-Seq is becoming an attractive approach in the profiling of gene expression and in evaluating differential expression [11].

However, RNA-Seq poses novel algorithmic and logistical challenges for data analysis and storage [12]–[15]. Despite the fact that many computational methods have been developed for alignment of reads, quantification of gene and/or transcripts, and identification of differentially expressed genes, there is great variability in the maturity of these available computational tools. In recent years, a large number of mapping algorithms [16]–[19] have been developed, and most recently, Engström et al [19] had performed a comprehensive assessment of the performance of 26 mapping protocols based on 11 programs and pipelines available, and found major performance differences between methods on numerous benchmarks, including alignment yield, basewise accuracy, mismatch and gap placement, exon splicing discovery and suitability of alignments for transcript reconstruction. They demonstrate that choice of alignment software is critical for accurate interpretation of RNA-Seq data, and identify aspects of the spliced-alignment problem in need of further attention.

The first step and a major challenge in RNA-Seq data analysis is the accurate mapping of sequencing reads to their genomic origins including the identification of splicing events. However, accurate alignment of high-throughput RNA-Seq data is a challenging and yet unsolved problem because of exon-eon spanning junction reads, relatively short read lengths and constantly increasing throughput of the sequencing technologies. Two key tasks make these analyses computationally intensive. The first task is an accurate alignment of reads that contain mismatches, insertions and deletions caused by genomic variations and sequencing errors. The second task involves mapping junction reads that span two or more exons. Although the first task is shared with DNA resequencing efforts, the second task is specific and crucial to the RNA-seq. These alignment challenges are further confounded by the presence of multiple copies of identical or related genomic sequences, making precise mapping difficult.

To detect splicing sites in short reads, there are three approaches published so far. The tools such as MapSplice [20] and TopHat [21] implement a two-step approach in which initial read alignments are analyzed to discover splicing sites; these splicings are then used to guide final alignments of those ‘initially unmapped reads’. However, this approach requires exons to have sufficiently high expression and will miss splicing events that are spanned by individual reads at a low level. The second solution for detecting splicing in short reads has been to align them to a reference transcriptome, possibly augmented with artificially constructed exon–exon segments. However, such an approach will identify only known or predicted combinations of exons, but not unexpected exon pairs that occur through exon skipping, cryptic splicing or gene fusions. In practice, these two approaches can be used in conjunction to improve placement of short sequence reads. Most recently, the “seed-and-vote” approach [22] is introduced. The new strategy uses a number of overlapping seeds from each read, called *subreads*. Instead of trying to pick the best seed, the strategy allows all the seeds to vote on the optimal location for the read. The algorithm then uses more conventional alignment algorithms to fill in detailed mismatch and indel information between the *subreads* that make up the winning voting block. The strategy is fast because the overall genomic location has already been chosen before the detailed alignment is done. It is also sensitive because no individual *subread* is required to map exactly, nor are individual *subreads* constrained to map close by other *subreads*.

Nowadays, many RNA-Seq aligners use reference transcriptome to inform spliced-read placements, including GSNAP [23], OSA [24], STAR [25], TopHat [21] and etc. In fact, this has become a common practice in RNA-Seq data analysis. However, no systematic evaluation has been performed to assess and/or quantify the benefits of incorporating reference transcriptome in mapping RNA-Seq reads. In this paper, we want to fill this gap. The same RNA-Seq dataset were first analysed with and without reference transcriptome, respectively, and then compared the mapping results and demonstrated the benefits for reference transcriptome in RNA-Seq data analysis.

## Methods

The Human Body Map 2.0 Project by Illumina generated RNA-Seq data for 16 different human tissues and can be accessible from ArrayExpress (accession # is E-MTAB-513). We used the 75 bp single read data from heart, liver, lung and kidney. In order to investigate the impact of sequencing depth on results, for each selected tissue, we sampled 10%, 30% and 60% of reads from the corresponding raw data, and analysed the subset data in the same protocol. The subsets are denoted as s10, s30, and s60, respectively (**Table 1**).

**Table 1:** Mapping summaries for all 4 samples and their subsets at different runs.

Samples	Total read#	RefGene/Unique		None/Unique		RefGene/Multiple		
		Uniquelymapped (%)	Unmapped (%)	Uniquelymapped (%)	Unmapped (%)	Uniquelymapped (%)	Non-uniquelymapped (%)	Unmapped (%)
heart	76,766,862	80.76	19.24	74.98	25.02	80.76	9.42	9.82
heart.s10	7,681,702	80.75	19.25	74.97	25.03	80.75	9.43	9.82
heart.s30	23,032,258	80.77	19.23	74.99	25.01	80.77	9.41	9.82
heart.s60	46,062,102	80.76	19.24	74.98	25.02	80.76	9.42	9.82
kidney	79,772,393	83.88	16.12	77.39	22.61	83.88	7.99	8.13
kidney.s10	7,982,115	83.88	16.12	77.39	22.61	83.88	7.99	8.13
kidney.s30	23,921,958	83.87	16.13	77.38	22.62	83.87	7.99	8.14
kidney.s60	47,863,118	83.88	16.12	77.39	22.61	83.88	7.99	8.13
liver	77,453,877	79.97	20.03	71.81	28.19	79.97	6.76	13.27
liver.s10	7,748,220	80.02	19.98	71.85	28.15	80.02	6.74	13.24
liver.s30	23,224,308	79.97	20.03	71.81	28.19	79.97	6.76	13.28
liver.s60	46,471,159	79.97	20.03	71.81	28.19	79.97	6.76	13.27
lung	81,255,438	88.65	11.35	79.21	20.79	88.65	4.50	6.85
lung.s10	8,125,846	88.64	11.36	79.19	20.81	88.64	4.51	6.86
lung.s30	24,376,159	88.65	11.35	79.20	20.80	88.65	4.50	6.85
lung.s60	48,750,504	88.65	11.35	79.21	20.79	88.65	4.50	6.85

The RefGene annotation files in GTF format was downloaded from UCSC genome browser on December 14 2012. First go to UCSC genome browser website (genome.ucsc.edu) and click “Table” menu to bring up “Table Browser” interface. Then set (1) genome to “human”, (2) assembly to “hg19”, (3) group to “Gene and Gene Predictions”, (4) track to “RefSeq Genes”, and (5) output format to “GTF – gene transfer format”. And then click “get output” button and save the text file. The RefGene model is used in our analysis.

Primary sequencing reads were first mapped to RefGene transcriptome and the human reference genome hg19 using OSA (Omicsoft Sequence Aligner, http://www.omicsoft.com/osa) [24], a super-fast and accurate alignment tool for RNA-Seq data. Benchmarked with existing methods such as Tophat and others, OSA improves mapping speed 4–10 fold with better sensitivity and less false positives. We have chosen OSA in *Stormbow* [26], a cloud-based pipeline we developed for large-scale RNA-Seq data analysis. OSA white paper (http://www.omicsoft.com/downloads/whitepaper/OmicsoftAligner.pdf) has more technical details on its implementation. For novel splicing sites (i.e. those junction sites not included in transcriptome), OSA uses Seed-Extend approach to discover junctions.

There are a total of three independent runs with different parameter settings: (1) **RefGene/Unique** run–aligning reads to RefGene transcriptome first and then to the reference genome, and only uniquely mapped reads are reported; (b) **None/Unique** run– reads are mapped to genome only without using reference transcriptome and only uniquely mapped read are kept. In this run; (c) **RefGene/Multiple** run–mapping reads to RefGene transcriptome and then to the genome, but reporting all locations if a read can be mapped equally well to multiple genomic regions. Next, we compared the difference of alignments for each read with and without RefGene transcriptome. All alignments were first exported into SAM text files [27], and then a Perl script was written to compare the alignments. Our comparison focused on “RefGene/Unique” versus “None/Unique”. For those reads mapped in “RefGene/Unique”, they can be divided into three categories according to their mapping results in “None/Unique” run. The first category is denoted as “**Identical**” – the mapping is exactly the same. The second is “**Alternative**” – still mapped but differently, either to a different genomic region or with different splicing sites. The last category is “**Unmapped**”, in which a mapped read becomes unmapped without the help of reference transcriptome.

We are particularly interested in those junction reads that spans two or more exons. Based upon the CIGAR [27] string for each mapped read in SAM files, we identified junction reads from each category, and calculated their statistical summaries. Additional analysis was performed on “Alternative” and “Unmapped” junction reads to characterize their splicing patterns in terms of the overlaps with exons. Furthermore, we investigated the key reasons for “Alternative” and “Unmapped” junction reads. At last, the “unique” versus “multiple” mapping mode was compared and explored.

## Results

### The overall mapping summary for different run settings

The mapping summary with different parameter settings was reported in **Table 1**. The column one corresponds to the total number of sequence reads. Compared “None/Unique” with “RefGene/Unique” run, an average of 6∼9% reads fail to be aligned. Clearly, with the help of RefGene model, more reads can be mapped, especially for junction reads as we demonstrated below. With respect to unique-mapping mode, roughly 5–10% more reads can be mapped in multiple-mapping mode, as we can see from the comparison of “RefGene/Multiple” with “RefGene/Unique”. We do not see much difference in mapping summary when a same sample is sequenced at varying depth in **Table 1**. Take the heart sample as an example; its uniquely mapped percentage in “RefGene/Unique” run is 80.76%, nearly identical to the results from its subsets in which 10%, 30% or 60% reads are randomly sampled, respectively.

As described above, all mapped reads in “RefGene/Unique” run can be divided into three categories with reference to their corresponding mappings in “None/Unique” run. After comparing the mapping results, the number of “Identical”, “Alternative” and “Unmapped” reads and their corresponding percentages are detailed in **Table 2**. While the majority of reads are not affected, 7.1 to 10.7% of mapped reads fail to be aligned without the help of RefGene model. Additionally, a small portion (ranging from 1.76 to 3.05%) of reads remain mapped but differently. As we demonstrate below, the alternative mapping is usually worse than its original mapping in “RefGene/Unique” run. It is noted that the impact of RefGene model on read mapping is sample dependant. As gene expression is very tissue specific, as a result, it is expected that each sample has its own unique gene expression profile. For the same sample, the effect of sequencing depth on the difference between “RefGene/Unique” and “None/Unique” runs is minimal or can be ignored according to **Table 2**.

**Table 2:** Summary of mapping difference between “RefGene/Unique” and “None/Unique” runs.

Sample	Total mapped reads	Identical	Alternative	Unmapped	Identical (%)	Alternative (%)	Unmapped (%)
heart.s10	5,693,483	5,190,188	100,105	403,190	91.16	1.76	7.08
heart.s30	17,073,146	15,563,618	300,572	1,208,956	91.16	1.76	7.08
heart.s60	34,142,552	31,124,065	600,018	2,418,469	91.16	1.76	7.08
heart	56,902,227	51,871,159	999,914	4,031,154	91.16	1.76	7.08
kidney.s10	6,294,377	5,674,231	134,507	485,639	90.15	2.14	7.72
kidney.s30	18,863,158	17,004,022	402,676	1,456,460	90.14	2.13	7.72
kidney.s60	37,748,639	34,026,386	805,300	2,916,953	90.14	2.13	7.73
kidney	62,910,741	56,708,369	1,342,853	4,859,519	90.14	2.13	7.72
liver.s10	5,650,928	4,946,767	126,339	577,822	87.54	2.24	10.23
liver.s30	16,926,181	14,820,387	378,023	1,727,771	87.56	2.23	10.21
liver.s60	33,869,672	29,654,325	756,300	3,459,047	87.55	2.23	10.21
liver	56,448,667	49,420,271	1,261,005	5,767,391	87.55	2.23	10.22
lung.s10	6,910,543	5,959,272	211,033	740,238	86.23	3.05	10.71
lung.s30	20,732,187	17,879,877	631,787	2,220,523	86.24	3.05	10.71
lung.s60	41,463,563	35,762,101	1,263,295	4,438,167	86.25	3.05	10.70
lung	69,112,294	59,606,596	2,105,556	7,400,142	86.25	3.05	10.71

The summary in **Table 3** is similar to **Table 2** but with a focus on junction reads only. According to **Table 3**, more than one third of junction reads cannot be aligned, and 10–15% reads are mapped in an alternative way when aligning reads without RefGene model. The “Identical” junction reads are only a little more than 50%. Once again, we do not see much effect of sequencing depth. Compared **Table 2** with **Table 3**, it is clear that junction reads are more vulnerable to mapping failure without the help of a gene model. In other words, reference transcriptome mainly impacts spliced-read placements.

**Table 3:** Summary of mapping difference for junction reads between “RefGene/Unique” and “None/Unique” runs.

Sample	Total junction read	Identical	Alternative	Unmapped	Identical (%)	Alternative (%)	Unmapped (%)
heart.s10	913,343	495,303	99,973	318,067	54.23	10.95	34.82
heart.s30	2,741,560	1,487,197	300,208	954,155	54.25	10.95	34.80
heart.s60	5,480,483	2,971,694	599,299	1,909,490	54.22	10.94	34.84
heart	9,138,263	4,955,529	998,728	3,184,006	54.23	10.93	34.84
kidney.s10	977,708	518,740	134,120	324,848	53.06	13.72	33.23
kidney.s30	2,930,648	1,554,577	401,485	974,586	53.05	13.70	33.25
kidney.s60	5,865,952	3,110,656	802,935	1,952,361	53.03	13.69	33.28
kidney	9,775,046	5,182,957	1,338,876	3,253,213	53.02	13.70	33.28
liver.s10	1,227,001	640,290	126,180	460,531	52.18	10.28	37.53
liver.s30	3,676,286	1,921,464	377,571	1,377,251	52.27	10.27	37.46
liver.s60	7,354,527	3,842,941	755,409	2,756,177	52.25	10.27	37.48
liver	12,262,340	6,406,568	1,259,511	4,596,261	52.25	10.27	37.48
lung.s10	1,418,039	725,789	210,746	481,504	51.18	14.86	33.96
lung.s30	4,251,055	2,175,156	630,859	1,445,040	51.17	14.84	33.99
lung.s60	8,506,036	4,356,543	1,261,446	2,888,047	51.22	14.83	33.95
Lung	14,176,505	7,260,245	2,102,444	4,813,816	51.21	14.83	33.96

**Figure 1:**
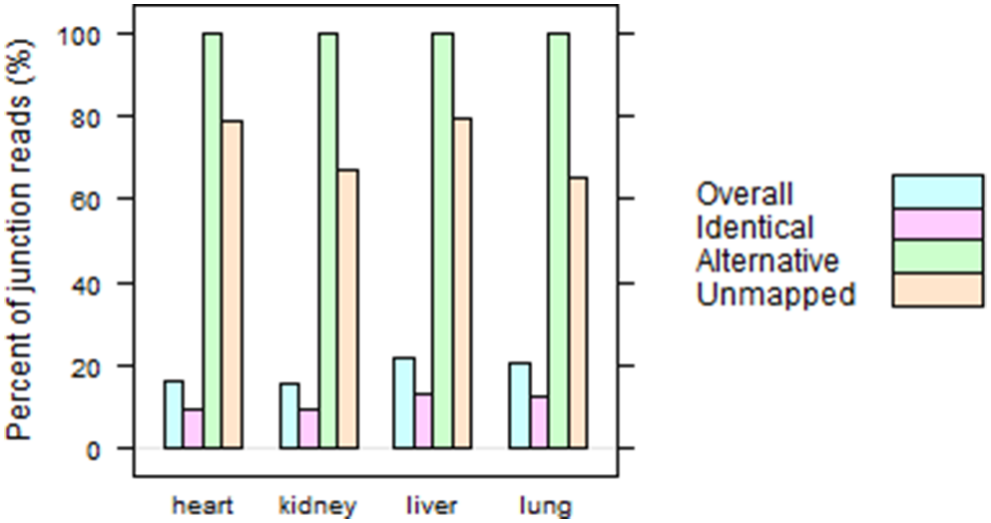
The percentages of junction reads over the total read in each category. Overall, about 20% mapped reads are junction ones. However, the percentages are nearly 100% and 65–80% for “Alternative” and “Unmapped” reads, respectively. RefGene transcriptome mainly influences the mapping of junction reads.

To highlight the impact of reference transcriptome on the mapping of junction reads, we calculated the percentages of junction reads over the total reads in each category (**Figure 1**) based upon the summary in **Table 2** and **Table 3**. Overall, junction reads account for about 20% of all mapped reads in “RefGene/Unique” run. However, the percentages jump up to nearly 100% for “Alternative” and 65–80% for “Unmapped” reads (**Figure 1**), respectively. This means nearly all “Alternatively” mapped and the majority of “Unmapped” in **Table 2** are junction reads. It is confirmed from **Figure 1** and **Table 3** that a reference transcriptome affects mainly the mapping of junction reads.

**Figure 2:**
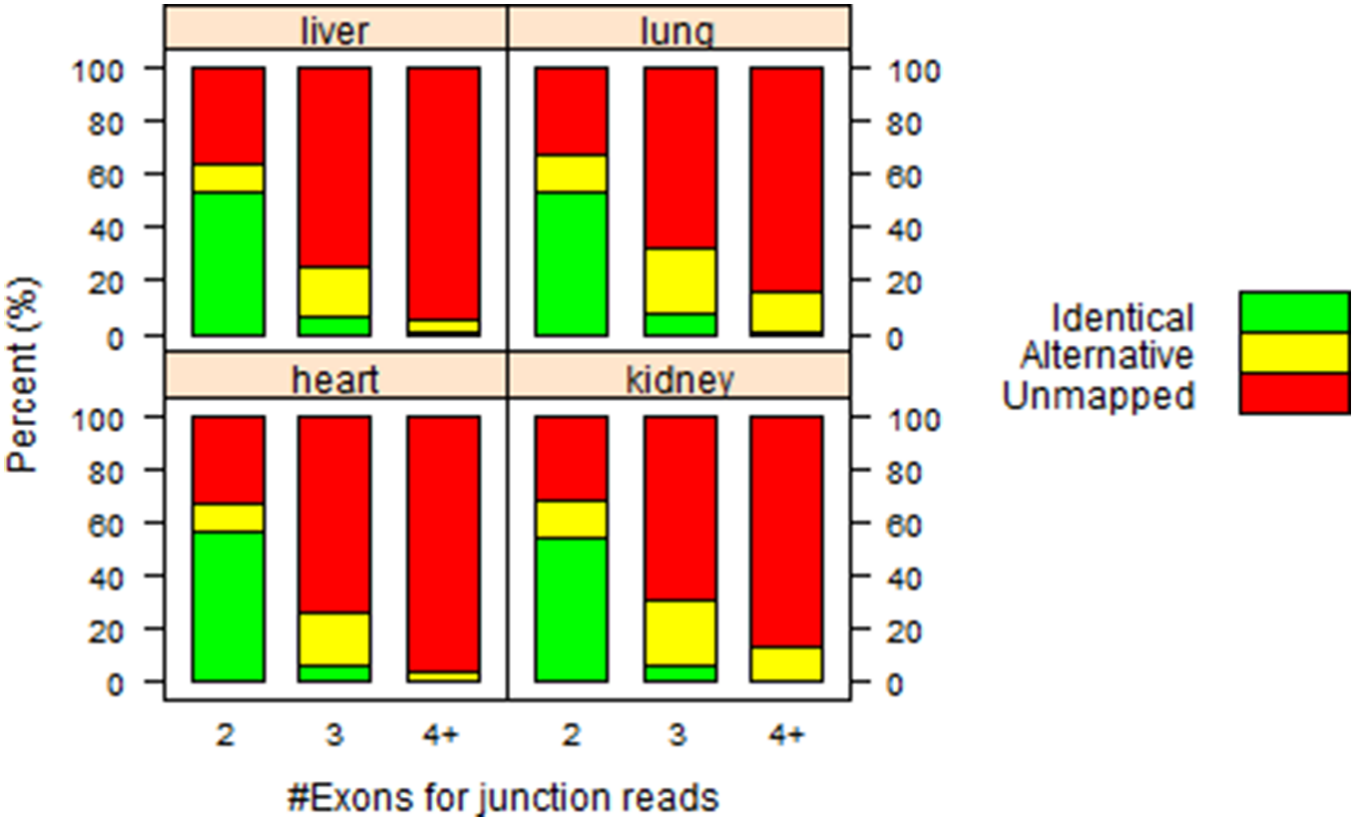
The relationship between the relative abundance for “Identical”, “Alternative” and “Unmapped” reads and the number of splicing sites. Evidently, the more exons a junction read spans, the less likely it can be mapped correctly in “None/Unique” run.

A junction read can span two or more exonic regions. Conceptually, the more exons a junction read spans, the harder it is to align it correctly without the help of a reference transcriptome. For those junction reads in **Table 3**, we further divided them into sub-groups according to the number of exons they span (see **Table 4**, **Figure 2**). On average, 55% two-exon junction reads can be aligned to the same genomic regions regardless of the usage of a gene model when mapping reads. For those junction reads spanning 3 exons, the percentage drops to less than 7%. And the percentage continues to drop to nearly zero for those junction reads spanning 4 or more exons. Obviously, the more exons a junction read spans, the less likely it can be mapped correctly without prior knowledge on junction sites in a gene model (**Figure 2**).

**Table 4:** Summary of mapping difference between “RefGene/Unique” and “None/Unique” for junction reads grouped by the number of exons spanned.

Sample	Exon number	Total Junction	Identical	Alternative	Unmapped
**heart**	2	8,790,698	4,933,717 (56.1%)	934,175 (10.6%)	2,922,806 (33.2%)
	3	337,724	21,798 (6.5%)	64,205 (19.0%)	251,721 (74.5%)
	4+	9,841	14 (0.1%)	348 (3.5%)	9,479 (96.3%)
**kidney**	2	9,459,854	5,163,154 (54.6%)	1,263,588 (13.4%)	3,033,112 (32.1%)
	3	311,641	19,775 (6.3%)	74,858 (24.0%)	217,008 (69.6%)
	4+	3,551	28 (0.8%)	430 (12.1%)	3,093 (87.1%)
**liver**	2	11,984,777	6,389,808 (53.3%)	1,205,425 (10.1%)	4,389,544 (36.6%)
	3	274,034	16,747 (6.1%)	53,904 (19.7%)	203,383 (74.2%)
	4+	3,529	13 (0.4%)	182 (5.2%)	3,334 (94.5%)
**lung**	2	13,686,975	7,225,169 (52.8%)	1,980,637 (14.5%)	4,481,169 (32.7%)
	3	479,948	35,040 (7.3%)	120,301 (25.1%)	324,607 (67.6%)
	4+	9,582	36 (0.4%)	1,506 (15.7%)	8,040 (83.9%)

### The splicing patterns for “Identical”, “Alternative” and “Unmapped” reads

**Figure 3:**
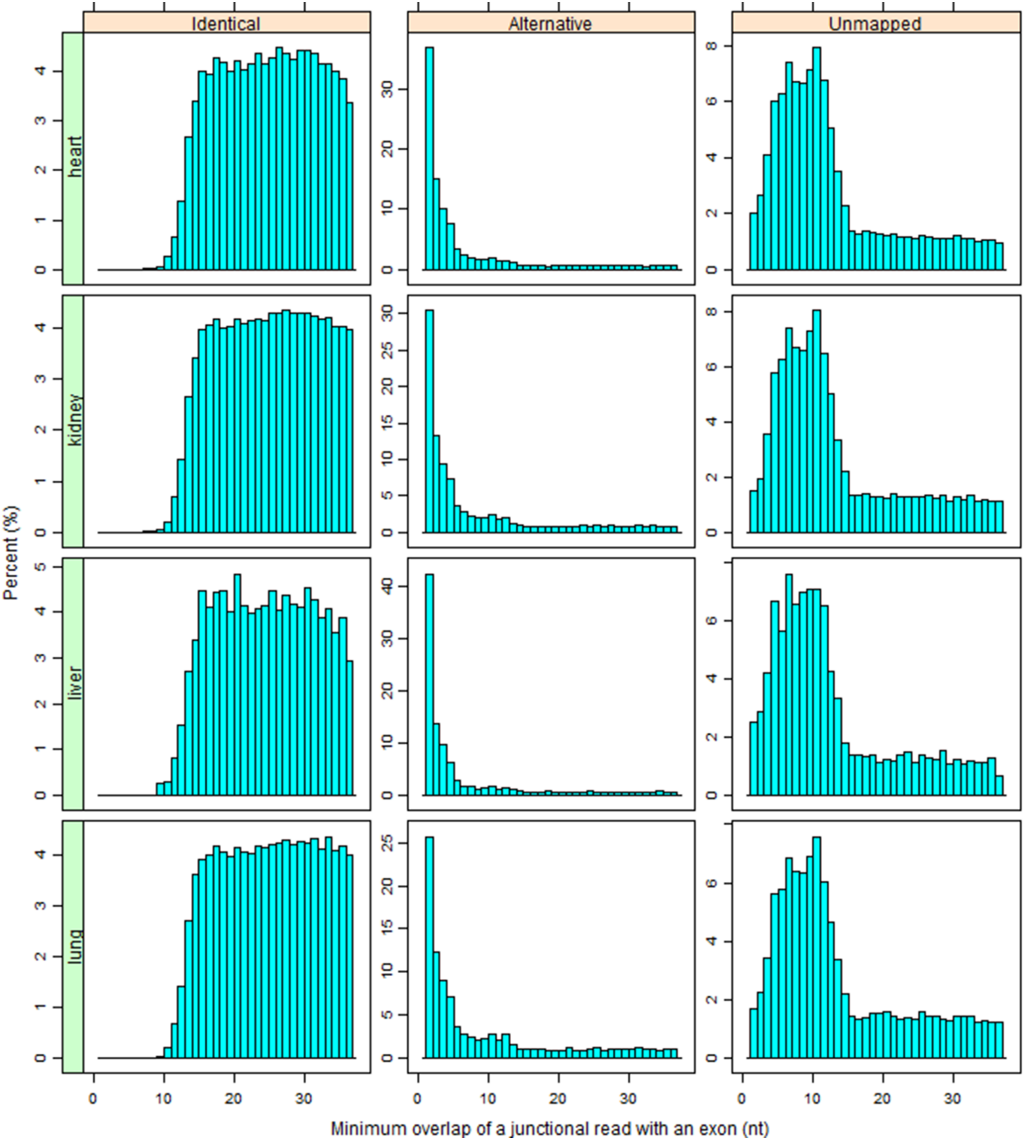
The distribution of MOE (Minimum Overlap with an Exon) for junction reads. The typical MOE for “Identical” junction reads ranges from 15 to 37. For “Alternative” junction reads, the most dominant MOE is 1, representing an average of one third of cases. In contrast, the MOE for “Unmapped” reads has a much broader range with peaks from 4 to 12. Note the scale for y-axis is not uniform.

As we conclude above, a reference transcriptome mainly affects the mapping of junction reads. For a junction read spanning more than two exons, it is hard to align it correctly without a reference transcriptome. One interesting question is what kind of junction reads tend to be mapped identically, alternatively or unmapped. According to **Table 4**, about 98% junction reads span only 2 exons. In order to characterize the splicing pattern, we focus on only two-exon junction reads. For each junction read in “RefGene/Unique” run, we calculate the number of overlapping nucleotide bases with its left exon (OL) and right exons (OR), respectively. Then the minimum of OL and OR is chosen for histogram analysis (**Figure 3**). Since the full read length is 75 bp long, the MOE (Minimum Overlap with an Exon, MOE = min (OL, OR)) ranges from 1 to 37 for any junction read. For “Identical” junction reads, the typical MOE ranges from 15 to 37, and the frequency drops to nearly 0 when MOE is less than 10. For “Alternative” junction reads, the most dominant MOE is 1, representing an average of one third of cases. In general, those “Alternative” reads have very small MOE. For those junction reads with MOE of 1, 2 and 3, it is virtually impossible to map them ‘correctly’ without the prior knowledge on transcripts. The MOE for “Unmapped” reads has a much broader range with peaks from 4 to 12. In order to map a junction read without a reference transcriptome, the read should have sufficient overlaps with exons at both ends. The majority of “Identical” reads meet this requirement (left panels in **Figure 3**). However, if the overlap with one end is too short, let’s say 1 or 2 nucleotide bases, this read will be more likely mapped to only a single exon with the remaining couple of bases mapping to the intron region adjacent to the exon (middle panels in **Figure 3**). Otherwise, such junction reads become either unmapped or mapped to different genomic regions as non-junction reads if the overlap is something between (right panels in **Figure 3**).

### Comparison of the mappings of “Alternative” reads

**Figure 4:**
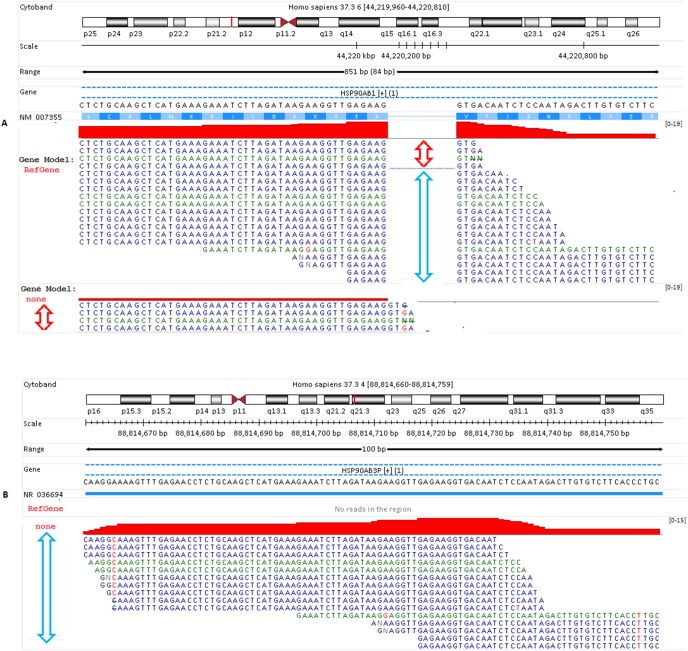
The impact of a reference transcripotome on the mapping of junction reads. (A) In “RefGene/Unique” run, 19 unique junction reads are mapped to gene HSP90AB1 nearly perfectly. Four of them remain mapped to the same gene but differently and with mismatches without the usage of RefGene model. In fact, the four junction reads become non-junction ones with a few bases mapped to the intron region; (B) The rest 15 reads (indicated by the blue arrow) are alternatively aligned to gene HSP90AP3P in “None/Unique” run, but with worse mismatches and alignment scores compared to their mappings in gene HSP90AB1 (**Figure 4A**). Note the reads colored in blue are mapped to “+” strand, and colored in green when mapped to “–” strand. The mismatch is colored in red.

Note that all the examples and illustrations below are from sample Heart.s10, and only unique reads are shown in genome browser. Since “Alternative” reads are mapped to either different genomic regions or splicing sites, we are more interested in the mapping difference in detail and the main reasons for alternative mapping. As shown in **Figure 4**, those 19 unique junction reads are nearly perfectly mapped to gene HSP90AB1 in “RefGene/Unique” run. Without a reference transcriptome, four reads indicated by red arrow remain mapped to the same gene HSP90AB1 but with mismatches at one end of the read. A few bases previously mapped to another exon are now mapped to the intron region, and accordingly the junction reads become non-junction ones now. The remaining 15 junction reads are aligned to pseudogene gene HSP90AP3P as non-junction reads instead. The comparison reveals that the original mappings to HSP90AB1 for those 15 reads are nearly perfect, while they all have more mismatches when mapped to HSP90AP3P. Clearly, the alternative mapping for those junction reads in **Figure 4** is getting worse without a reference transcriptome. In a sense, those 15 junction reads indicated by blue arrow in **Figure 4A** are “forced” to be mapped to a different genomic region without the help of reference transcriptome. Usually, such ‘forced’ mappings have mismatches, and quite often unusual coverage pattern is seen, as we will see later.

**Figure 5:**
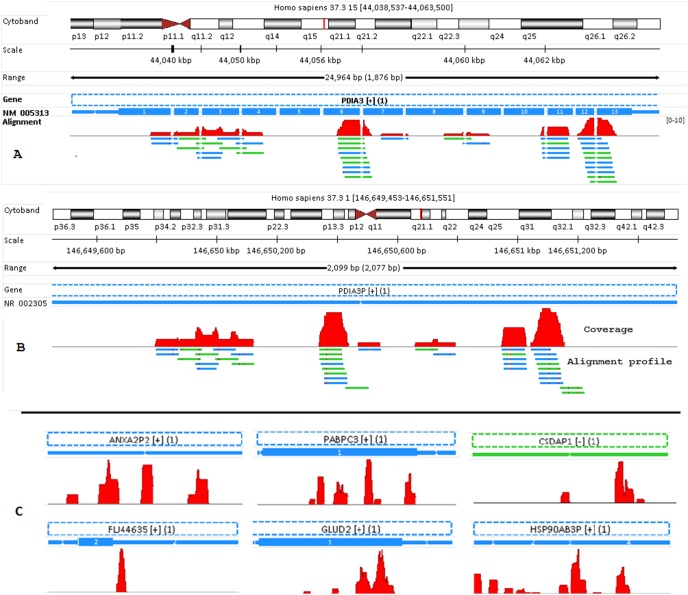
False mappings of “Alternative” junction reads. (A) With RefGene model, these 38 reads are uniquely mapped to 8 splicing regions in gene PDIA3, and the alignments are nearly perfect. None of them is mapped to PDIA3P. (B) Without a gene model, however, all those 38 reads are uniquely mapped to retrotransposed pseudogene PDIA3P as non-junction reads. All alignments have 1 or two mismatches (indicated by red dots in alignment profile). (C) Without a gene model, all sequences mapped to these six genes are non-junction reads. They are re-mapped to elsewhere as junction reads with the usage of RefGene model. The read coverage profiles in **Figure 5B** and **Figure 5C** are neither flat nor continuous, and those sporadic coverage patterns are quite unusual. If these genes in **Figure 5B** and **Figure 5C** are truly expressed, we should usually see mapped reads across the entire exon regions, not just a few sporadic and isolated islands. Due to falsely mapped reads, we get misleading information on the expressions of these genes. Note gene CSDAP1 and its transcript is encoded in “–” strand, and colored in green. All the rest other genes colored in blue since they are encoded in “+” strand.

The impact of ‘Alternative’ junction reads on gene quantification is demonstrated and further illustrated in **Figure 5**. With the help of RefGene model, these 38 junction reads in **Figure 5A** are uniquely mapped to 8 splicing regions in gene PDIA3, and the alignments are nearly perfect. None of them is mapped to PDIA3P. However, all those 38 reads are uniquely mapped to retrotransposed pseudogene PDIA3P instead as non-junction reads without a gene model. Note in **Figure 5B** no other reads are mapped to PDIA3P. All alignments in **Figure 5B** have 1 or two mismatches (indicated by red dots in alignment profile). The island-like read coverage pattern in **Figure 5B** also indicates those mapped reads are false positives. If gene PDIA3P is truly highly expressed, ideally we should see mapped reads along exons evenly. Another sign for false mapping is mismatch, as indicated by those tiny red dots in alignment profile in **Figure 5B**. Each dot represents a single mutation or mismatch with respect to its nucleotide base in reference genome.

Those 38 sequence reads in **Figure 5A and 5B** are interesting. They are junction reads in the transcriptome when mapping them with the help of RefGene model (**Figure 5A**), but all become non-junction ones in the genome without a gene model (**Figure 5B**). The scenario illustrated in **Figure 5A and 5B** occurs when a gene has a retrotransposed pseudogene copy elsewhere in the genome. This is the case of PDIA3 (parent gene) and PDIA3P (retrotransposed pseudogene). Retrotransposed pseudogene is generated by reverse transcription of an mRNA transcript with subsequent reintegration of the cDNA into the genome, and the processed pseudogenes can also accumulate random disablements such mutations over the course of evolution. That’s why those mapped reads in **Figure 5B** have mismatches. The island pattern we observe in **Figure 5B** corresponds to the reads that should have been mapped to the junctions of the parent gene PDIA3, but have wrongly been mapped to the retrotransposed pseudogene PDIA3P. As a result, those falsely mapped reads in **Figure 5B** can give rise to misleading information on the expression of pseudogene PDIA3P. In the meantime, we also underestimate the expression of the parent gene PDIA3.

Similarly, for all those genes shown in **Figure 5C**, neither read remains mapped to them in “RefGene/Unique” run. Like **Figure 5B**, the sporadic island-like coverage patterns in **Figure 5C** do not support that those mapped reads truly originate from there either. As a matter of fact, ANXA2P2 is a processed pseudogene, and its parent gene is ANXA2. HSP90AB3P is a retrotransposed pseudogene as well, and it parent gene is HSP90AB1. Those sequences mapped to HSP90AB3P should have been mapped to HSP90AB1 as junction reads, as shown in **Figure 4**. Without the usage of a reference transcriptome, we get misleading expressions for those genes in **Figure 5C** as well due to ‘falsely’ mapped reads, and simultaneously underestimate the expression of those genes to which the reads should have been mapped.

**Figure 6:**
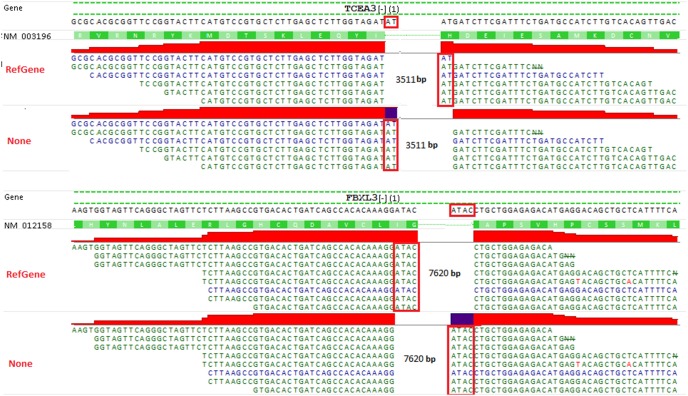
Alternative splicing in “RefGene/Unique” and “None/Unique” runs. All junction reads are still mapped to the same gene. The start/end positions and intron size are exactly the same regarding of gene model, but split differently. Without the assistance of reference transcriptome, we cannot tell which mapping is correct. Note the coverage profile corresponding to an exon and an intron are colored in red and dark blue, respectively.

“Alternative” junction reads are also likely to be mapped to the same start and end positions but splitting at different sites. Two cases in point are shown in **Figure 6**. For those junction reads mapped to gene TCEA3 with and without RefGene model, both mappings are equally well in terms of alignment scores and gaps between exons. So there is no way to tell which one is right without the assistance of reference transcriptome. Likewise, the mappings of junction reads in gene FBXL3 are also equally well regardless of the usage of RefGene model. Despite the minor difference in splicing sites, the read mapped with RefGene model is considered as fully compatible to a known gene, and thus is counted in gene quantification. However, without a gene model, the same read is mapped to exon-intron, and thus it is discarded at quantification step when only fully compatible reads are counted. For some counting packages, such as HTSeq [28], whether such reads partially overlapping with exons are counted is dependent upon the setting of the overlap resolution mode. When the mode is set to *intersection-strict* in HTSeq, the reads mapped to exon-intron are excluded. Thus, the alternative mappings in **Figure 6** are not equal at gene quantification step in RNA-Seq data analysis.

### Investigation on “Unmapped” reads

**Figure 7:**
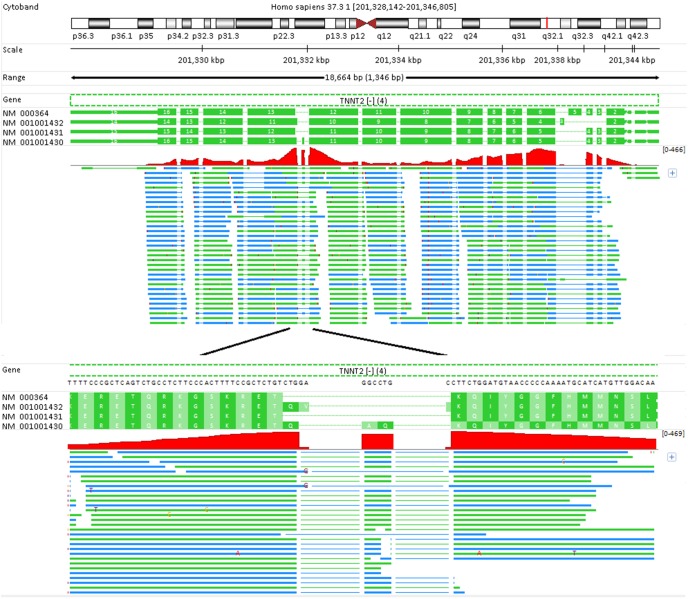
Junction reads across small exons can be mapped with the help of RefGene model, but not without the assistance of a reference transcriptome. The region around exon 12 in the transcript NM_001001430 is zoomed out (the bottom panel). This exon is too short, only 6 bp long, and there is no way to map a junction read to so small an exon without prior knowledge on splicing sites. Note reads are colored in blue if mapped to “+” strand, and green if mapped to “–” strand.

More than one third of junction reads fail to be aligned without the help of RefGene model (see **Table 3**). As shown in **Figure 3**, “Unmapped” junction reads tend to have a short 4–14 bp overlap with one of exons. Without the help of a reference transcriptome, it is hard to find the right splicing sites, especially for those reads spanning very small exons. Gene TNNT2 is a good example (**Figure 7**). There are 4 annotated transcripts derived from this gene by alternative splicing. Most exons are short, and even shorter than the read length. Note the exon 12 in the transcript NM_001001430 is as short as 6 bp long, and there is no way to map a junction read to such a small exon without prior knowledge (see bottom in **Figure 7**). There are a total 2,016 unique junction reads mapped to this gene in “RefGene/Unique” run. Without a gene model, 2000 of those junction reads cannot be mapped to this gene anymore. Accordingly, the expression level for this gene is significantly underestimated due to the mapping failure of the majority of junction reads. This also happens for other genes such as MYH6, MYH7, TPM1, KTN1 and RPL9, in which hundreds or thousands of junction reads become unmapped without the help of a reference transcriptome.

**Figure 8:**
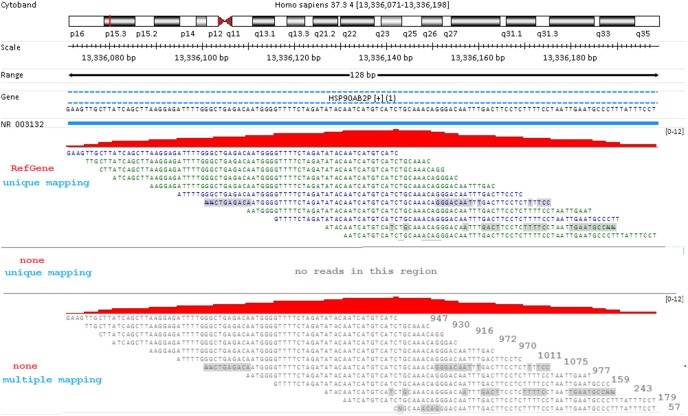
The mapping of non-junction reads in different mapping mode. Twelve reads are uniquely mapped to HSP90AB2P with RefGene model in unique mapping mode (top panel), but all fail to be mapped in “None/Unique” run (middle panel). It turns out these reads can be mapped to multiple genomic regions in addition to gene HSP90AB2P, and thus discarded in “None/Unique” run. The total number of mapping positions across the genome for each read is shown to the right of each sequence (bottom panel). The 7^th^ sequence can be mapped to as many as 1,075 positions.

As we can see from **Figure 1**, about 70% “Unmapped” reads are junction ones, and the rest ∼30% are non-junction reads. Why is the mapping of a non-junction read affected by a reference transcriptome? We are puzzled by this phenomenon at first sight. After careful investigation, we realized that the main reason is mapping mode. A read that is uniquely mapped in RefGene model is likely to become a multi-read when mapping across the genome, and accordingly excluded when only uniquely mapped read is reported. In **Figure 8**, there are 12 reads uniquely mapped to gene HSP90AB3P in “RefGene/Unique” run, unfortunately those 12 non-junction reads become unmapped in “None/Unique” run. When multiple-mapping is enabled and the threshold for reported mapping locations is set to 2000, those 12 reads can be mapped to multiple genomic regions, ranging from 57 to 1,075. At the bottom of **Figure 8**, the number to the right of each sequence is the number of genomic regions where this read can be mapped to. **Figure 8** illustrates a scenario in which a unique read in a reference transcriptome can become a multi-read if mapped genome wide. Therefore the usage of reference transcriptome can reduce the mapping ambiguity for some RNA-Seq reads.

Those “Unmapped” reads in **Figure 7** and **Figure 8** have dramatic impacts on accurate estimation of gene expression levels. In general, the expression is underestimated. We have quantified the effect of those “Unmapped” junction reads in **Table 3** on gene expression, and it is found that there are a total 700 genes (see **Table S1**) in which the read counts are reduced by 20% or more due to the mapping failure of junction reads without the help of reference transcriptome.

### Unique-mapping versus multiple-mapping

**Figure 9:**
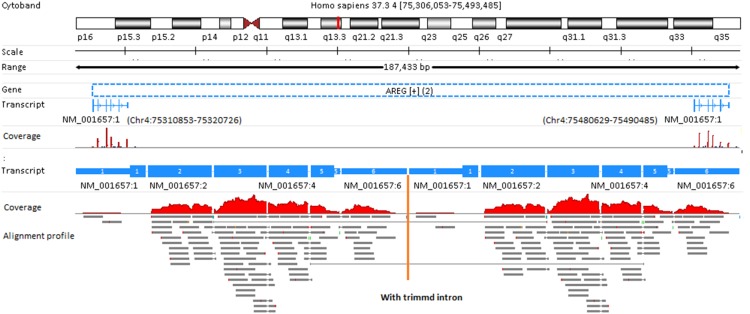
The necessity of multiple-mapping mode for gene AREG. Transcript NM_001657 can be generated by alternative splicing from two genomic regions. All reads mapped to one region are surely to be mapped to its twin region as well. As a result, these reads are excluded in unique-mapping mode. In order to properly map these multi-reads to gene AREG, multiple-mapping mode is necessary.

A read can be mapped to multiple locations not only across the reference genome, but even within a reference transcriptome. For instance, in the case of recent paralogs, reads obtained by the sequencing of one member of the gene family will usually map to several members. Gene AREG is an extreme example where the gene is present in 2 copies that did not diverge (**Figure 9**). These two identical transcripts are located at genomic regions Chr4: 75310853–75320726 and Chr4: 75480629–75490485, respectively. The transcript consists of 6 exons. At the bottom of **Figure 9**, introns are trimmed and the alignment profiles are shown. Note the alignment profile on the left is exactly identical to the alignment profile on the right. Each read in alignment profile is colored gray, meaning it is a multi-read. In multiple-mapping mode, the expression level for this gene is 20.23 RPKM, while in unique-mapping mode, the expression is ZERO. Obviously, unique-mapping mode is inappropriate for gene AREG.

**Figure 10:**
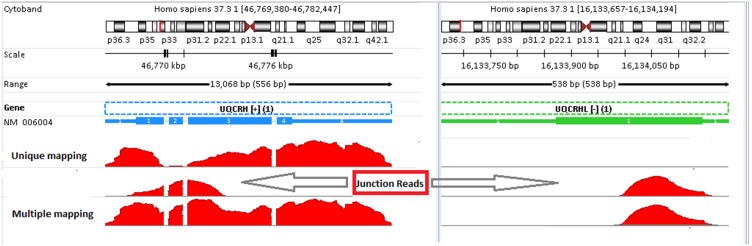
The impact of mapping mode on gene quantification: unique-mapping versus multiple-mapping mode. Those junction reads on the track between “Unique mapping” and “Multiple mapping” tracks are mapped to the junction regions around exon #2 in gene UQCRH, as well to gene UQCRHL. As a result, these reads are excluded in unique-mapping mode. Consequently, we have a very shallow read coverage around exon #2 in gene UQCRH when aligning reads in unique-mapping mode. In contrast, in multiple-mapping mode, those reads are mapped, and accordingly, the read coverage profile for gene UQCRH significantly improves (left bottom).

Another interesting example is shown in **Figure 10** to demonstrate the impact of mapping mode on gene quantification. In unique-mapping mode, very few reads are mapped to the regions around exon #2 in gene UQCRH. While in multiple-mapping mode, the read coverage profile is more like a flat terrain. Intuitively, multiple-mapping mode works better than unique-mapping mode in term of the pattern of coverage profile. Those reads mapped to gene UQCRH in multiple-mapping mode but not in unique-mapping mode turn out to be junction reads around exon #2, as shown on the track between “Unique mapping” and “Multiple mapping” tracks in **Figure 10**. In addition to gene UQCRH, those junction reads can be mapped to gene UQCRHL equally well. Therefore, they are multi-reads in RefGene model. That is why they fail to be mapped to gene UQCRH in unique-mapping mode. Note in **Figure 10** if we map RNA-Seq reads without a reference transcriptome and in unique-mapping mode, those junction reads are mapped to gene UQCRHL only (right in **Figure 10**). As a consequence, gene UQCRHL is reported to have a high expression level, and this is untrue. If gene UQCRHL is indeed highly expressed, we should be able to see reads mapped to other regions of this gene as well. The example is **Figure 10** illustrates the importance of both a reference transcriptome and the multiple-mapping mode in accurate mapping RNA-Seq reads.

According to the mapping summary reported in **Table 1**, about 5∼10% more reads are mapped in “RefGene/Multiple” run compared with “RefGene/Unique” run, If we increase the reporting threshold for multi-reads from 10 to a higher number, let’s say 100, more non-uniquely mapped reads are expected in **Table 1**. The impact of mapping mode on alignment is sample dependent (**Table 1**). As we know, a significant challenge in analyzing RNA expression of homologous genes is the large fraction of the reads mapped to multiple locations in the reference transcriptome and genome. In unique-mapping mode, all multi-reads are discarded, and this is too stringent and not ideal for those genes in **Figure 9** and **Figure 10**. As previously noted [1], if the multi-reads are discarded, the expression levels of genes with homologous sequences will be artificially deflated. Our research has demonstrated the necessity of multiple-mapping, and thus, in conjunction with reference transcriptome, multiple-mapping mode is strongly recommended for RNA-Seq data analysis. When a read is mapped to multiple locations, report all locations instead of randomly pick one of the locations. The tools for downstream gene quantification can decide whether to include or how to assign multiple-mapping reads to those mapped genes or transcripts.

### The impact of a reference transcriptome on differential analysis

**Figure 11:**
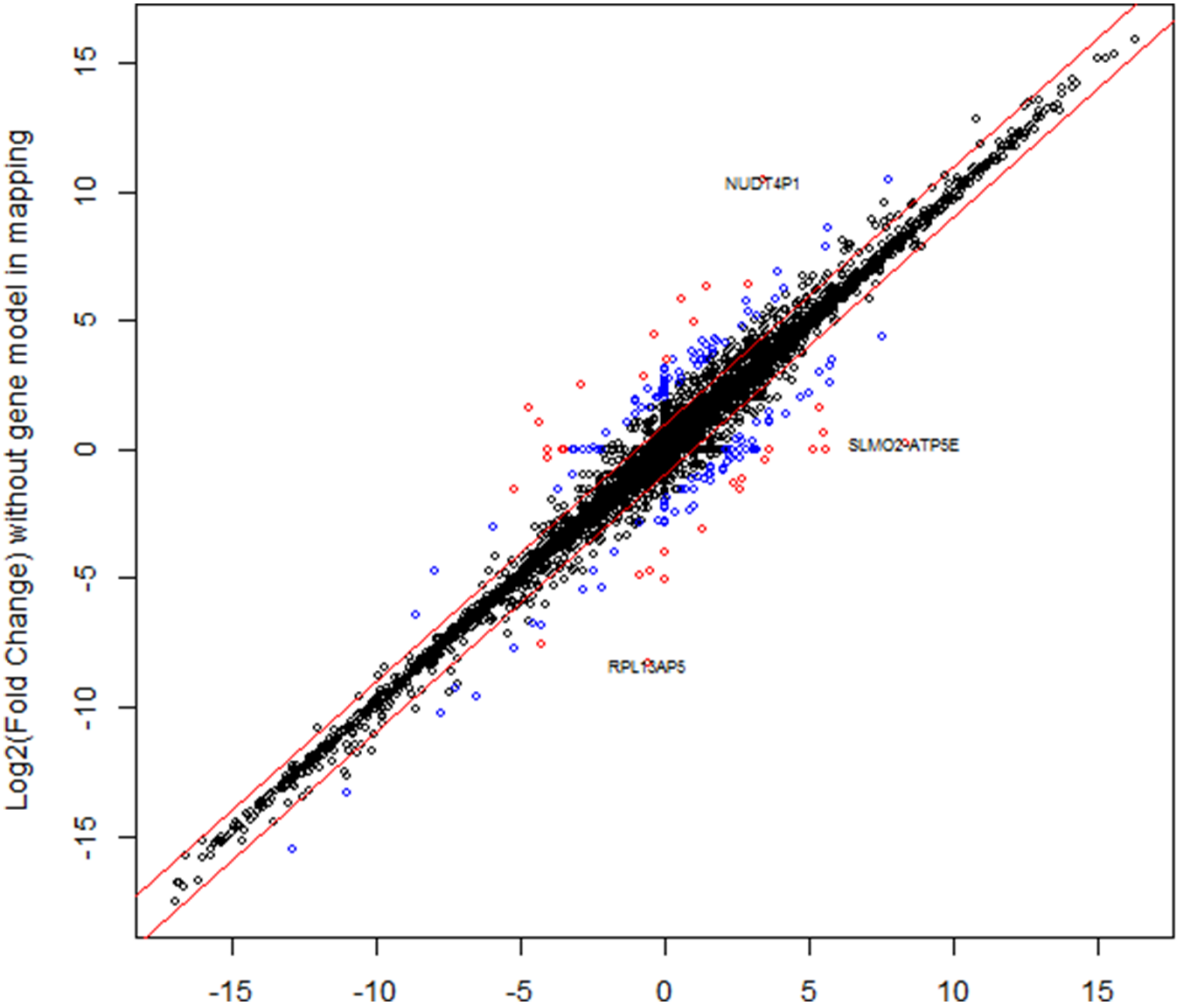
The correlation of the calculated Log2(Fold Change) between heart and liver samples with and without the usage of RefGene transcriptome in mapping. The points were colored in blue and red, respectively, if the corresponding absolute difference between the two Log2(Fold Changes) was greater than 2 or 3.3. Although the majority of genes had highly consistent expression changes, there were quite a few genes that were dramatically affected by the choice of using transcriptome reference or not.

Usually, RNA-Seq differential analysis requires replicates. However, we have a single sample in each different tissue. To demonstrate the impact of a reference transcriptome on differential analysis, we calculated the fold change between heart and liver samples with and without the usage of RefGene transcriptome in mapping. The correlation of the calculated Log2(Fold Change) was shown in **Figure 11**. Ideally, we should get a straight line if the reference transcriptome has no impact on differential analysis. Obviously, this is not true. Although the majority of genes had highly consistent or comparable expression changes, there were quite a few genes that are dramatically affected by the choice of using transcriptome reference or not. The points were colored in blue and red, respectively, if the corresponding absolute difference between the two Log2(Fold Changes) was greater than 2 or 3.3. Some genes were found to have an expression change greater than 32-folds (2∧5) in one analysis, but was found not to change in the other analysis. Clearly, using reference transcriptome in read mapping has an impact on the downstream differential expression analysis.

## Discussion

Short reads generated by RNA-Seq experiments must ultimately be aligned, or “mapped” to a reference genome or transcriptome assembly. The general objective when mapping a collection of sequencing reads to a reference is to discover the true location (origin) of each read with respect to that reference. Read alignment to a reference provides biological information in two basic ways. First, it generates a dictionary of the genomic features represented in each RNA-Seq sample. Second, the number of reads aligned to each feature approximates abundances of those features in the original sample. Such measures of digital gene expression are then subject to comparison among samples or treatments in a statistical framework. Despite several years of ongoing improvements, alignment of the junction RNA-Seq reads to a reference genome is not a solved problem yet, owing both to its intrinsic complexity and rapid advances in the sequencing technologies. Thus the accuracy of read mapping and gene quantification is critical for differential analysis.

In this paper, we have studied the impact of reference transcriptome on mapping RNA-Seq reads, especially on junction reads. As shown in **Table 3** and **Figure 1**, only about 50–55% junction reads can be mapped to the same genomic regions regardless of the usage of RefGene model. More than one third of junction reads fail to be mapped without the help of a reference transcriptome. For “Alternative” mapped reads, their mappings in “None/Unique” run are usually worse than their corresponding alignments in “RefGene/Unique” run, as we demonstrated **in** **Figure 4** **,** **5** and **6**. For those junction reads spanning more than two exons (**Table 4** and **Figure 2**), it is less likely to correctly align them without the help of reference transcript.

All sequence reads in our dataset are 75 bp long. It is noted that the results in this paper will be different if the same tissue sample are sequenced at other different read lengths. The impact of the read length on the mapping of junction reads remains an interesting question for our exploration in the future. Nowadays, most providers, including BGI (the largest sequence service provider in the world), offer RNA-Seq sequencing service to customers by delivering 50–150 bp short reads. As the sequencing technology evolves, the read length is becoming longer and longer. For instance, the newest MiSeq desktop sequencer from Illumina and its MiSeq Reagent Kits v3 can generate reads of 300 bp long. Read length certainly has a remarkable effect on the detection of exon-exon junction and on the mapping of exon-exon spanning reads. When reads become longer, they are more likely to span multiple exons, and thus the mapping of long junction reads is actually becoming more and more challenging without the assistance of reference transcriptome. Thus, the need to have reference transcriptome included in the mapping is greatly increased. Therefore, the advantages of using reference transcriptome in the mapping demonstrated in this study are becoming more evident for longer reads. Indeed, long read lengths increase read uniqueness in mapping, but make alignment of junction reads more difficult without the prior knowledge on transcripts.

However, when the reference transcriptome used to guide the mapping of reads is incomplete or inaccurate, some biases or errors can be introduced as well. Pyrkosz et al [29] has explored the issue of “RNA-Seq mapping errors when using incomplete reference transcriptome” in detail. They used simulated reads generated from real transcriptomes to determine the accuracy of read mapping, and measured the error resulting from using an incomplete transcriptome. When 10% increments of the chicken reference transcriptome are missing, the true positive rate decreases by approximately 6–8%, while the false positive rate remains relatively constant until the reference is more than 50% incomplete. The number of false positives grows as the reference becomes increasingly incomplete. For model organisms such as human and mouse, their transcriptome models are relatively more complete compared to non-model organisms. The human RefGene transcriptome used in our analysis is a collection of non-redundant, curated mRNA models. It is a relatively stable reference for genome annotation, gene identification and characterization, mutation and polymorphism analysis, expression studies, and comparative analyses. Admittedly, RefGene transcriptome is not 100% complete and accurate, but its quality is constantly improving. For transcriptome guided mapping of RNA-Seq reads, the more complete and accurate the transcriptome, the better.

Pyrkosz et al [29] also notice that the completeness of the reference transcriptome interacts significantly with the mapping mode. The true positive rate for multiple-mapping mode is dependent solely on the correct transcript being in the reference, while the unique-mapping mode has substantially lower true positive rates. This is consistent with our results. In conjunction with reference transcriptome, it is highly recommended to map RNA-Seq reads in multiple-mapping mode for both junction and non-junction reads, as we have demonstrated in **Table 1**, **Figure 9** and **Figure 10**.

In addition, Seok et al [30] have demonstrated that incorporating transcript annotations from reference transcriptome significantly helps the de novo reconstruction of novel transcripts from short sequencing reads for transcriptome research. The prior knowledge helped to define exon boundaries and fill in the transcript regions not covered by sequencing data. As a result, the reconstructed transcripts were much longer than those from de novo approaches that assume no prior knowledge. From the same RNA-Seq dataset in [29], Velvet, an assembly algorithm based on de Bruijin graphs [31], reconstructed transcripts with a median length of 207 bases, and Trinity [14], another de novo algorithm that does not rely on aligning reads to a reference genome, reported a median length of transcripts of 173 bases, which are much shorter than the median length 1,553 bases from the algorithm developed by Seok et al with transcript annotations in RefSeq as prior knowledge. These results corroborate the usefulness of leveraging reference transcriptome not only in mapping of junction reads, but also in the reconstruction of novel mRNA transcripts from sequencing data.

## Supporting Information

Table S1 **A total 700 genes in which the read counts are reduced by 20% or more due to the mapping failure of junction reads without the assistance of reference transcriptome.**
(XLSX)
